# Wenyang Huazhuo Tongluo formula, a Chinese herbal decoction, improves skin fibrosis by promoting apoptosis and inhibiting proliferation through down-regulation of survivin and cyclin D1 in systemic sclerosis

**DOI:** 10.1186/s12906-016-1056-6

**Published:** 2016-02-20

**Authors:** Li Han, Hua Bian, Jingfeng Ouyang, Yuefeng Bi, Lei Yang, Songshan Ye

**Affiliations:** Zhang Zhongjing College of Chinese Medicine, Nanyang Institute of Technology, Changjiang Road 80, Nanyang, 473004 Henan China; Experimental Research Center, China Academy of Chinese Medical Sciences, Beijing, China; School of Pharmaceutical Sciences, Zhengzhou University, Zhengzhou, Henan China

**Keywords:** Wenyang Huazhuo Tongluo formula, Systemic sclerosis, Anti-fibrosis, Survivin, Cyclin D1

## Abstract

**Background:**

Fibrosis is a major contributor to systemic sclerosis (SSc)-related morbidity, and rapid, progressive skin involvement predicts later mortality. Western medicine therapies for SSc cannot produce satisfactory effects currently, while Traditional Chinese Medicine (TCM), such as the Wenyang Huazhuo Tongluo (WYHZTL) formula, a Chinese herbal decoction, has shown amazing anti-fibrosis efficacy on SSc in clinical applications. This study is aiming to investigate the anti-fibrotic mechanism of WYHZTL formula for the treatment of SSc.

**Methods:**

Fibroblasts from primary culture of skin lesions of SSc patients were exposed to rat medicated sera containing WYHZTL or XAV939, a small-molecule inhibitor of both tankyrase 1/2 and Wnt/β-catenin pathway. Cell counting kit-8 assay and Annexin V FITC/PI apoptosis kit were used to analyze cell proliferation and apoptosis in fibroblasts, respectively. Reverse transcription-polymerase chain reaction (RT-PCR) and western blotting were used to detect the mRNA and protein levels of cyclin D1 and survivin.

**Results:**

After 28, 48 and 72 h of incubation, the proliferative ability of the fibroblasts cells was obviously reduced by the sera containing WYHZTL compared with that in the control group; the percentage of apoptotic cell population in the sera containing WYHZTL treated fibroblasts cells was significantly higher than that in those treated with the control sera, and was about similar to that in those treated with XAV939. The sera containing WYHZTL could down-regulate both mRNA and protein levels of cyclin D1 and survivin, compared with the control group.

**Conclusions:**

The present study demonstrates the antiproliferative and pro-apoptotic actions of WYHZTL formula against fibroblasts and the effect may be related to the down-regulation of mRNA and protein levels of cyclin D1 and survivin in SSc.

## Background

Systemic sclerosis (SSc) is an autoimmune and/or autoinflammatory disease mainly characterized by the excessive accumulation of extracellular matrix (ECM), especially as collagen, and by extensive fibrosis in skin and eventually in multiple internal organs, such as lung, heart, esophagus and kidney [1]. Genetic factors, infection and unknown environmental influences may play roles in the pathogen of the disease. However, the exact initiating events that lead to the disease remain unclear. Immune dysfunction, vascular damage, abnormal metabolism of connective tissue and a complex interaction of them are thought to be involved in SSc. Fibrosis is a major contributor to the disease related morbidity, and rapid, progressive skin involvement predicts later mortality [2]. Western medicine therapies mainly include immunosuppressive agents, vasoactive drugs and connective tissue formation inhibitors. However, the clinical effects of these therapies are of questionable significance currently, such as methotrexate and cyclophosphamide [3, 4].

Considering the unsatisfactory effect of current therapies for SSc, it would be desirable to have novel drugs/therapies. Traditional Chinese medicine (TCM) has a long history of dealing with diseases and evolved over the past two and a half millennia to become the second largest health-care system in the world, after modern Western medicine. In the past few years, much research has focused on the molecular mechanisms of TCMs to treat different diseases, for example, Shuang Kou et al. [5] reported that Zuo-Gui and You-Gui pills exerted neuroprotective effects by downregulation of NogoA, NgR, and RhoA pathways in rats with experimental autoimmune encephalomyelitis. Some effective TCM formulas have also been found to treat SSc through acting on certain molecular targets. Yan et al. [6] reported that Wenyang Chubi Decoction had the effects in decreasing the connective tissue growth factor and collagen-I expression and improving the skin fibrosis in a mouse model of SSc. Yiqihuoxue formula was found to effectively reduce collagen production via down-regulating the phosphorylation of Smad3 and then the activity of Smad binding element, which are involved in the TGF-beta pathway and constitutively activated in the progression of SSc [7].

Wenyang Huazhuo Tongluo (WYHZTL) is a patented formula (No. CN201310351880.2) to treat SSc owned by our team, and has shown the efficacy of anti-fibrosis in clinical applications. Our previous studies proved that it exerts its therapeutic effect on SSc patients by regulating Th17/Treg imbalance, lowering levels of von Willebrand factor (vWF) and aminoterminal propeptide of type III procollagen (PIIINP), and elevating the level of cross-linked carboxyterminal telopeptide of type I collagen (I CTP) [8]. We also reported that the formula antagonizes the fibrosis in a mouse SSc model through its regulation of TGF-β1 /Smad signal pathway and the reduction of collagen content [9]. We also have demonstrated that the sera containing WYHZTL reduces the expression levels of collagen I/III through regulating some key signal molecules, such as TGF-β1 receptor I, p-Smad2/3, Smad7, in TGFβ1/Smad Signaling pathway of skin fibroblasts obtained from SSc patients [10]. In another report, we found the sera containing WYHZTL inhibits the proliferation of SSc skin fibroblasts via blocking the cell cycle transition from the G1 to S phase [11], but the exact mechanism is still unclear. Therefore, further study on the role of WYHZTL in fibrosis of SSc is needed. It’s known that the cell cycle transition from the G1 to S phase is regulated by cyclin D1, and cyclin D1 activation promotes the passage of cells through the G1 restriction checkpoint [12, 13]. Apoptosis is a key mechanism involved in fibrosis of SSc [14], and survivin is an inhibitor of apoptotic protein gene family and a cell cycle regulator, it is critically required for suppression of apoptosis and ensuring normal cell division in the G2/M phase of the cell cycle [15].

In order to address the anti-fibrotic mechanism of WYHZTL formula in treating SSc, a novel seropharmacology method arising recent years for pharmacological study is taken to study TCM in vitro using animal-medicated sera [16]. The essence of seropharmacology is to administrate TCM to experimental animals (generally are rabbits or rats), followed by harvesting animal sera after a period of time. Then the sera are applied to different cell lines for molecular mechanism study. The advantage of seropharmacology for TCM study is to use the real pharmaceutical ingredients or internal biological metabolic compounds of TCM, without isolating the individual components, which is neither difficulty nor necessary in some cases.

The aim of the present study is to investigate the mechanism of WYHZTL formula against fibrosis in SSc regarding its effect on the expression and regulation of cyclin D1 and survivin in primary cultured human fibroblasts treated by the animal-mediated sera.

## Methods

### Composition and preparation of Wenyang Huazhuo Tongluo formula

The ingredients of WYHZTL formula were purchased from the First Affiliated Hospital of Nanyang Institute of Technology and the full ingredients are *Radix Astragali membranacei*, *Herba Epimedii*, *Ramulus Cinnamomi cassiae*, *Herba Glechomae longitubae*, *Semen Sinapis albae*, *Radix Dioscoreae oppositae*, *Radix Codonopsitis pilosulae*, *Radix Rehmanniae praeparata*, *Fasciculus vascularis Luffae and Capparis zeylanica Linn*. All of the ingredients of WYHZTL were prepared as crude slices and boiled twice (90 min per time) with ultrapure water as the doctor’s directions. The water extracts were combined, filtered and evaporated under reduced pressure to a final concentration of 1.5 g/mL based on the equivalent amount of the crude drugs.

### Cell culture

Skin biopsy specimens were obtained from five progressive SSc patients, who had the disease course of less than 2 years and were fulfilled the American College of Rheumatology criteria for SSc [17]. Biopsy specimens (3 mm × 3 mm) were performed at the edge of the lesion of each SSc patient. All patients provided written contents, and the study was approved by the Ethics Committee of Nanyang Institute of Technology, China.

Skin samples were transported in DMEM supplemented with 10 % fetal bovine serum (FBS), 100 IU/mL penicillin, and 100 mg/mL streptomycin for processing the same day. The skin samples were washed in 75 % ethanol, phosphate buffered saline (PBS), and the DMEM supplement. Cultured fibroblasts were established by mincing tissues and placing them into 60-mm culture dishes secured by glass coverslips. Third-to sixth-passage fibroblasts were used for gene and protein expression assays.

### Preparation of rat medicated sera

The rat medicated sera was prepared according to the published protocols [18]. Briefly, 30 Wistar female rats, aged between 6 and 8 weeks old and weighing 220 ~ 250 g, were divided into WYHZTL (*N* = 15) and control (*N* = 15) groups. The animals were supplied by the Henan Experimental Animal Center (Zhengzhou, China) and maintained in accordance with accepted standards of humane animal care as outlined in ethical guidelines for care and use of laboratory animals in an air-conditioned room with controlled a temperature of 22 ± 2 °C, a humidity level of 45 % to 65 %, and a 12/12 h light/dark cycle. The rats in WYHZTL group were individually administrated by gavage with WYHZTL decoction of 6.25 g/kg/day based on clinical dosage. The control group received deionized water. After 10 days of administration, blood was collected from retinal venous plexus and centrifuged. The collected sera were aliquoted into 5 mL Eppendorf tubes and preserved at-80 °C for future use.

### Sera samples preparation for HPLC analysis

One milliliter of the rat medicated sera were mixed with 5 mL of methanol and ultrasonicated for 20 min. The mixture was then centrifuged at 3000 rpm for 10 min at room temperature, and the supernatant was evaporated to dryness under a steam of nitrogen at 50 °C. The residues were reconstituted in 100 *μ*L of methanol and filtrated through a 0.45 *μ*m membrane filter (Millipore Co. Ltd., Tokyo, Japan), and an aliquot (20 *μ*L) was applied to the HPLC analysis. The HPLC analysis was performed with a Waters Alliance 2695 HPLC system. The HPLC conditions were as follows: column: Agilent C18 column (150 mm × 4.6 mm); column temperature: 30 °C; mobile phases: 0.1 % ammonium acetate (A) and linear gradient system of methanol (B), where A/B: 85/15 (0 min), 70/30 (5 min), 30/70 (10 min), 30/70 (16 min), and 85/15 (28 min); flow rate: 1 mL/min; detection wavelength: 259 nm; and injection volume: 20 *μ*L.

### Proliferation assay

Cell proliferation was analyzed with the cell counting kit-8 (CCK-8; Santa Cruz Biotechnology, USA) assay. Briefly, the fibroblast cells were harvested in exponential growth and seeded into 96 well plates at a density of 1.0 × 10^4^ cells per well. The supernatant was removed after cell adhered to the wall and added FBS-free DMEM medium to incubate another 24 h. And then the cells were treated with the rat medicated control sera, the WYHZTL sera or XAV939 (final concentration 2 ng/mL; Sigma-Aldrich), a small-molecule inhibitor of tankyrase 1/2 and Wnt/β-catenin pathway, plus the WYHZTL sera respectively. The sera volume ratio for incubation is 15 %. After incubated 24, 48 and 72 h, CCK-8 was added into the cells at a volume rate 1:10 to incubate for 2.5 h. The absorbance was measured at 450 nm using an enzyme immunoassay analyzer (Dynatech MR4100, USA). The proliferation rate was calculated for each well. The mean and standard deviation of five relative proliferation rates for each well were calculated. The cell morphology was observed under an inverted microscope during the experimentation.

### Measurement of apoptosis by flow cytometry

Analysis of apoptosis percentage was performed using an Annexin V-FITC/PI apoptosis kit (KeyGen Biotech, China) as previously described protocol [19]. Briefly, the cells were incubated with the rat medicated control sera, the WYHZTL sera and XAV939 plus the WYHZTL sera respectively for 24, 48 and 72 h. The sera volume ratio is same as above. When reaching 80 % confluence, cells were trypsinized, washed with PBS and resuspended in 500 μl of Annexin V-binding buffer. A mixture of 5 μl of FITC-labeled Annexin V plus 5 μl of Propidium Iodide (PI) solution was then added into the cells to incubate for 15 min at room temperature in the dark prior to flow cytometry analysis. Cell apoptosis was analyzed using CellQuest software (Becton-Dickinson). Three separate experiments were performed for each clone.

### Western blot analysis

The effects of the rat medicated sera containing WYHZTL on cyclin D1 and survivin protein expression was analyzed as previously described protocol with slightly modification [20]. Briefly, the fibroblast cells were treated with the sera as above at a density of 1.0 × 10^9^ cells per 25 ml culture flask for 24, 48 and 72 h, and then the cells were lysed using RIPA lysis buffer (Beyotime Institute of Biotechnology, China). The protein concentrations were determined using the bicinchoninic acid assay (BCA; Santa Cruz Biotechnology, USA). A total of 40 μg of proteins was electrophoresed via sodium dodecyl sulphate polyacrylamide gel electrophoresis (SDS-PAGE), and transferred onto polyvinylidene fluoride (PVDF) membranes (Millipore, USA), which were then blocked with 5 % skimmed milk powder for 1 h at room temperature. The membranes were incubated with antibodies against cyclin D1 and survivin (1: 1000 dilution; Santa Cruz Biotechnology, USA), followed by incubation with corresponding horseradish peroxidase (HRP)-conjugated secondary antibody at a 1:5000 dilution for 1 h at 37 °C . Finally, the membranes were washed with PBS three times and the immunoreactive bands were visualized using an ECL-PLUS/Kit according to the manufacturer’s instructions. The optical density of each band was measured with a computer-assisted imaging analysis system (Quantity One, Bio-Rad, Hemel Hempstead, UK) and the relative protein levels were normalized to optical density of β-actin.

### RT-PCR analysis

Total RNA from individual groups was extracted using the TRIzol reagent (Invitrogen, USA). All RNA preparation and handling steps took place in a laminar flow hood, under RNAse-free conditions. The isolated RNA from each fraction was dissolved in 20 *μ*L of RNAse-free water and stored at-80 °C until used. cDNA synthesis was performed at 37 °C for 15 min and 85 °C for 5 sec using the Primer Script RT reagent Kit (TaKaRa Biotechnology, Dalian, China) in a total volume of 20 *μ*L according to the manufacturer’s instructions. The forward and reverse primers for amplifying cyclin D1 are as follows: 5′-CGATGCCAACCTCCTCAAC-3′ and 5′-AAGCCTTGTAATCCTTGTG-3′, and the length of the amplified fragment was 212 bp. The forward and reverse primers for amplifying survivin were as follows: 5′-GGACCACCGCATCTCTACA-3′ and 5′-GCACTTTCTTCGCAGTTTCC-3′, and the length of the amplified fragment was 338 bp. A 196-bp fragment of GAPDH was used as the internal control and was amplified with the following forward and reverse primers: 5′-GAGTCAACGGATTTGGTCGT-3′ and 5′-GACAAGCTTCCCGTTCTCAG-3′. The PCR cycling conditions comprised a denaturation step for 5 min at 95 °C, followed by 30 cycles of denaturation (94 °C for 15 s), annealing (55 °C for cyclin D1, 54 °C for survivin and 57 °C for GAPDH for 30s), and extension (72 °C for 30s). After the last cycle, all PCR products were subjected to a final extension for 10 min at 72 °C. PCR products were combined and then electrophoresed on 1.5 % agarose gels containing ethidium bromide; the images were captured using a Gel Doc XR+ Image Station (Bio-Rad, Hemel Hempstead, UK). The optical density of each band was measured with Quantity One and normalized to optical density of the housekeeping gene GAPDH.

### Statistical analysis

Data were analyzed with SPSS 16.0. Statistical data are expressed as mean ± SD. Group comparisons were performed using one-way ANOVA followed by a Tukey test for multiple comparisons. The results were considered statistically significant when *P* < 0.05. Graphic representation was performed using GraphPad Prism version 6.01 for Windows (GraphPad Software, San Diego CA, USA, www.graphpad.com).

## Results

### Analysis of the compounds absorbed into the blood after oral administration of WYHZTL decoction in SD rats

**Fig. 1:**
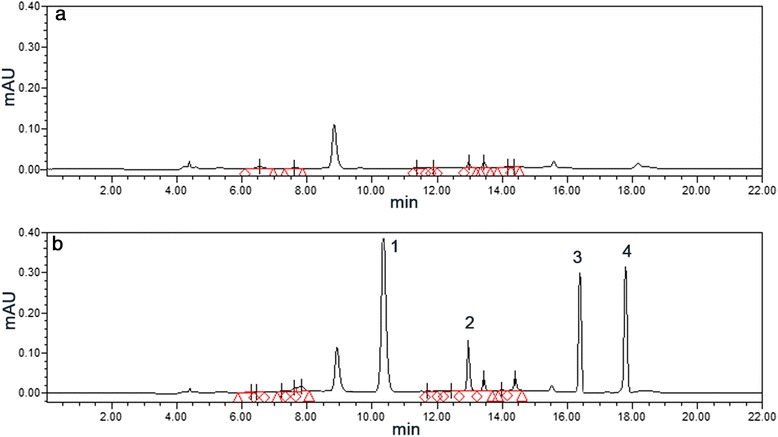
HPLC profiles of the rat medicated sera after administrated by gavage of the WYHZTL decoction. **a** Control rat sera; **b** rat medicated sera after administrated by gavage 1 h. Four distinctive peaks (1, 2, 3 and 4) are detected in the rat medicated sera, but not in the control rat sera. This indicates that the rat medicated sera may contain active ingredients and/or metabolic components of the WYHZTL decoction

Figure 1 shows the representative HPLC profiles of the control rat sera and the rat medicated sera, respectively. Four distinct peaks can be detected in the rat medicated sera containing WYHZTL compared with the control rat sera, which indicates that the rat medicated sera may contain active ingredients and/or metabolic components of the decoction.

### Effect of rat medicated sera containing WYHZTL on proliferation of fibroblasts

**Fig. 2:**
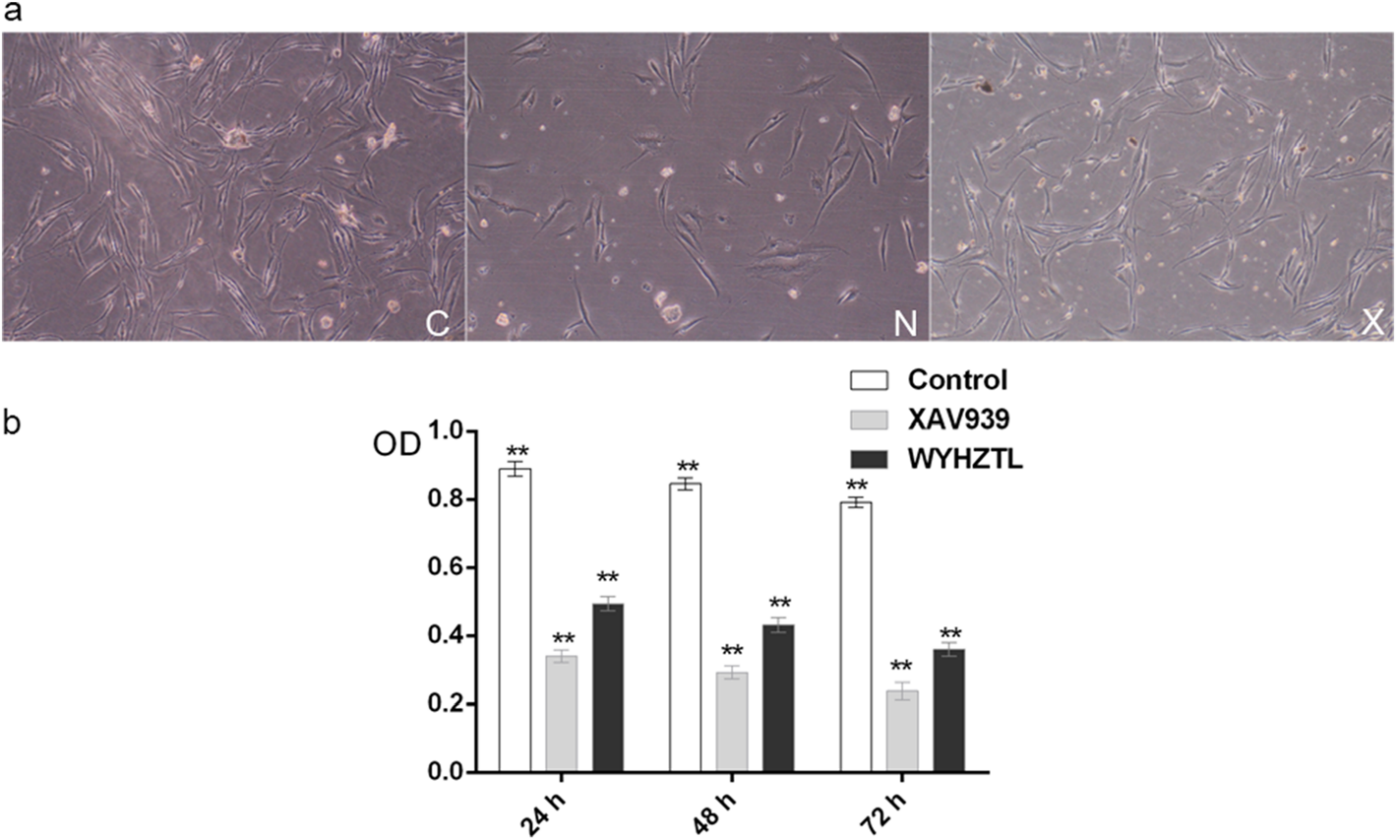
Effect of Rat Medicated Sera containing WYHZTL on proliferation of fibroblasts. **a** Cell morphology observation: cells cultured in DMEM containing the sera were grown in the flask and viewed directly under the Nikon Phase Contrast Inverted Microscopy (200× original magnification; Ti-S type, Nikon Co., Japan). **b** The CCK-8 assay was performed to examine cell proliferation. The absorbance measured at 450 nm by using an enzyme immunoassay analyzer (Dynatech MR4100, USA) after incubated for 24, 48 and 72 h. The mean and standard deviation of 5 relative proliferation rates for each well were calculated. C represents control group, N represents XAV939 inhibitor group, X represents rat medicated sera containing WYHZTL group. **P < 0.01 compared with each group

In order to confirm the role of WYHZTL formula in the proliferation of fibroblasts, the rat medicated sera containing WYHZTL were added into the primary culture fibroblasts. The morphology of the cells was observed directly under an inverted microscope. In the control group, fibroblasts were numerous and having branched cytoplasm surrounding elliptical, speckled nucleus with two or more nucleoli. Most nucleoli were spindle and some were irregular, but all tended to be in the same direction. After incubated with XAV939 or the sera containing WYHZTL, both the total cell and the irregular cells with multi-nucleoli decreased, the cell bodies, nucleoli and the branched cytoplasm were shorter, compared with those in the control group (as shown in Fig. 2a). Such results demonstrated that the sera containing WYHZTL strongly implicated in cell morphology regulation and had inhibitory effect on proliferation in fibroblasts.

The above evidence was further confirmed by CCK-8 assay. After incubated with the rat medicated sera or XAV939 for 28, 48 and 72 h, the proliferative ability of the fibroblasts cells was markedly reduced and was significantly lower than that in the control group following the indicated time of incubation (*P* < 0.01), as shown in Fig. 2b.

### Rat medicated sera containing WYHZTL promotes apoptosis in fibroblasts

**Fig. 3:**
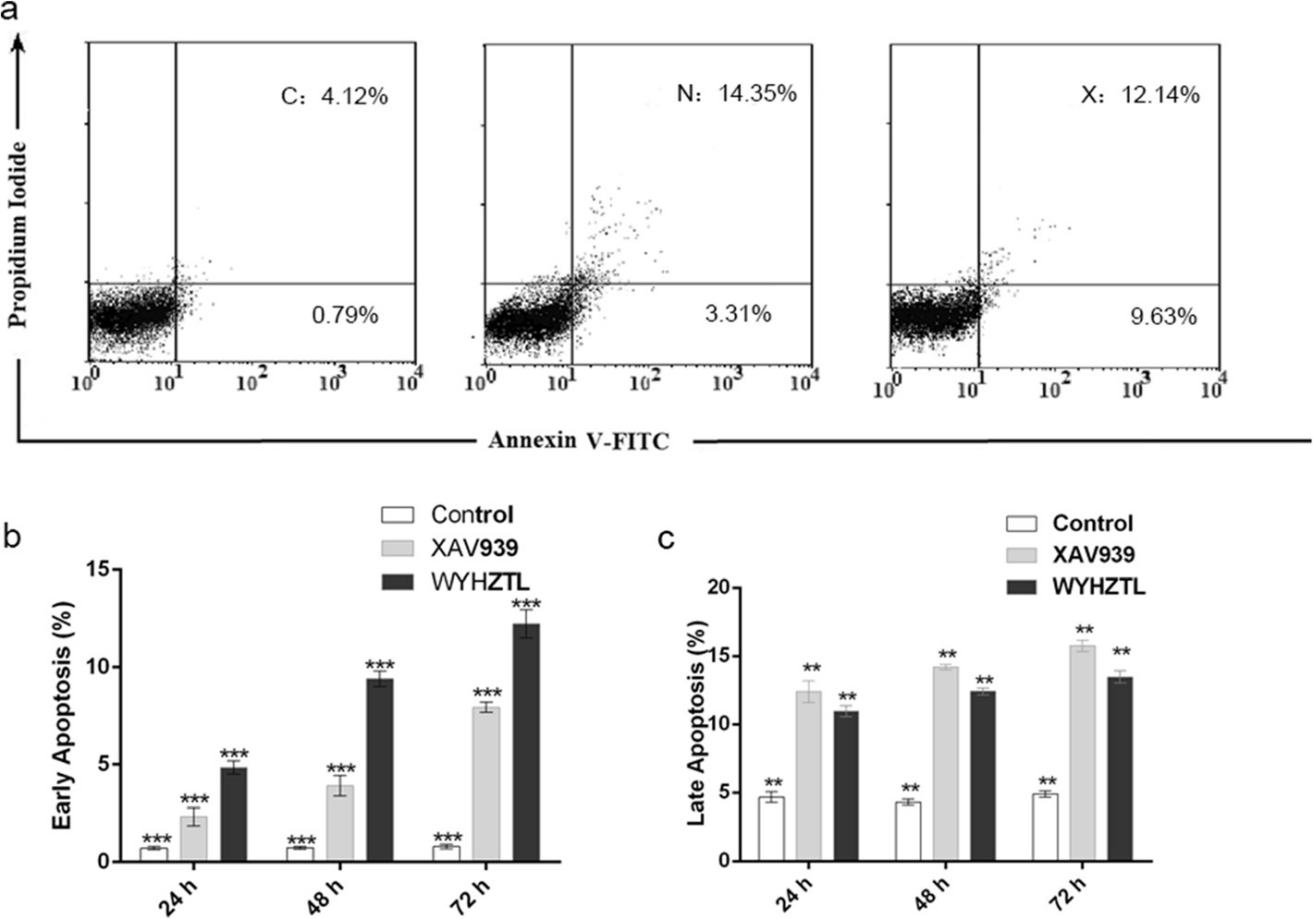
Rat medicated sera containing WYHZTL induces cell apoptosis in fibroblasts. **a** Cell apoptosis was examined after incubation for 48 h by Annexin V-PI staining using flow cytometry. Representative results are shown. **b** Bar graph of early apoptosis rate after incubated with the sera or XAV939 for 24, 48 and 72 h. **c** Bar graph of late apoptosis rate after incubated with the sera or XAV939 for 24, 48 and 72 h. C represents control group, N represents XAV939 inhibitor group, X represents rat medicated sera containing WYHZTL group. **P < 0.01, ***P < 0.001 compared with each group. The results are the mean ± SD of 3 independent experiments

In order to determine the effects of the rat medicated sera containing WYHZTL on apoptosis in fibroblasts, viable, early apoptotic, and late apoptotic or necrotic cells can be distinguished by flow cytometry analysis using dual staining with Annexin V/PI dyes. As shown in Fig. 3a, viable cells were PI and Annexin V-FITC double negative; apoptotic cells were Annexin V-FITC positive and PI negative, whereas late apoptotic or necrotic cells were Annexin V-FITC and PI double positive. We observed a strong time-dependent relationship with regard to the rat medicated sera containing WYHZTL or XAV939 exposure and early apoptotic or late apoptotic cells (Fig. 3b, c). The percentage of late apoptotic cell population in the sera containing WYHZTL treated cells was clearly higher than that in those treated with the control sera and was slightly lower than that in those treated with XAV939 (*P* < 0.01). Interestingly, the percentage of early apoptotic cell population in the sera containing WYHZTL treated cells was clearly higher than that in those treated with XAV939 (*P* < 0.01), which suggesting the rat medicated sera containing WYHZTL promotes apoptosis, especially early apoptosis, in the fibroblasts. This effect was also observed in XAV939 treated cells.

### Effect of rat medicated sera containing WYHZTL on cyclin D1 in fibroblasts

**Fig. 4:**
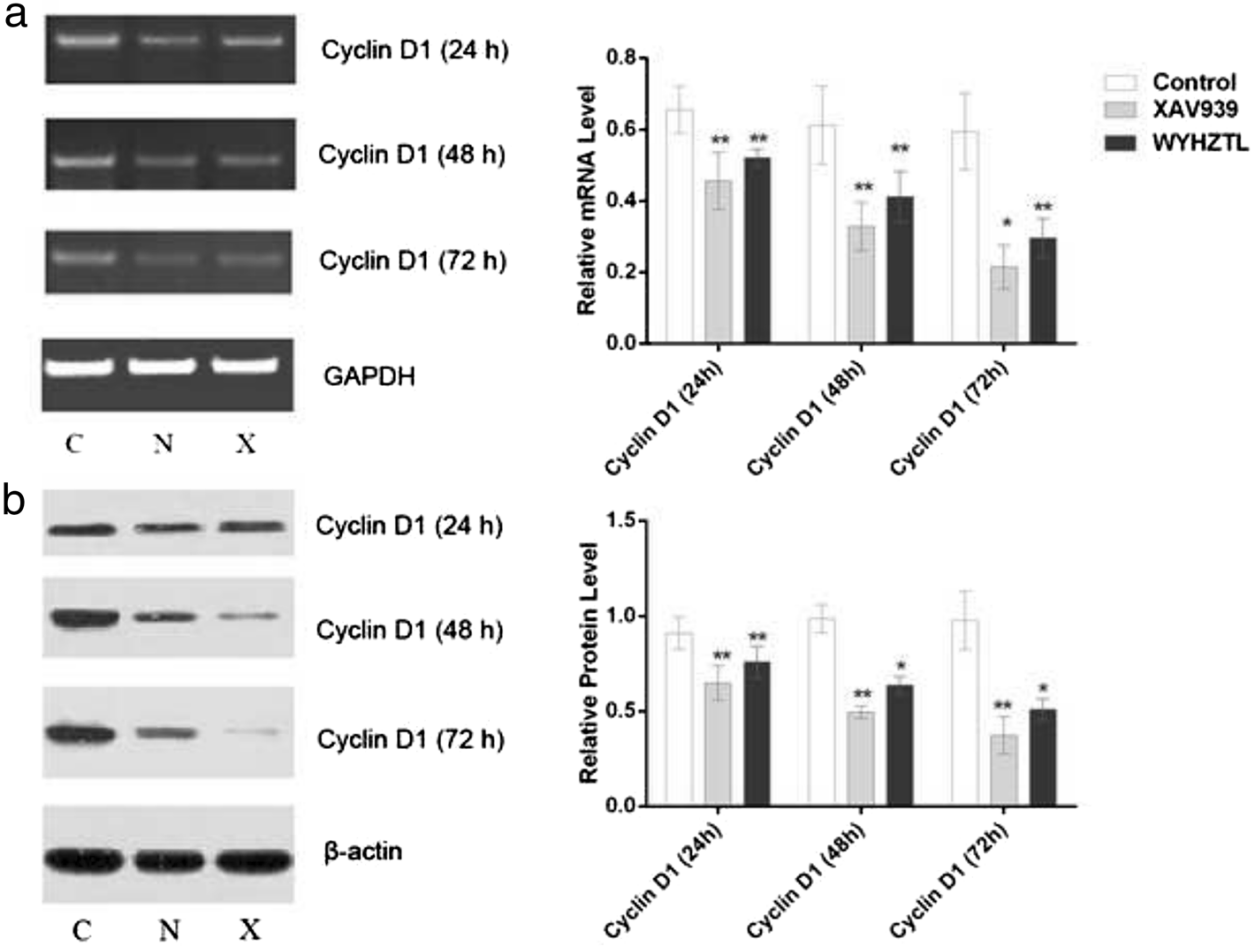
Effect of down-regulation of cyclin D1 by the rat medicated sera containing WYHZTL in fibroblasts. **a** The mRNA levels of cyclin D1 were detected by RT-PCR after treated with the sera containing WYHZTL for 24, 48 and 72 h. GAPDH was applied as an endogenous control and densitometric values were normalized by GAPDH. **b** The protein levels of cyclin D1 were detected by Western blotting after treated with the sera containing WYHZTL for 24, 48 and 72 h. β-actin was used as an endogenous control and densitometric values were normalized by β-actin. Representative results are shown. C represents control group, N represents XAV939 inhibitor group, X represents Rat Medicated Sera containing WYHZTL group. Densitometric values are shown as mean ± SD. Left panels represent RT-PCR/Western blotting gel results, right panels represent bar graphs for relative mRNA and protein levels for cyclin D1. *P < 0.05, **P < 0.01, compared with the control group. The results are the mean ± SD of at least 5 independent experiments

In order to further explore the molecular mechanisms of the role of the rat medicated sera containing WYHZTL, we focused on the recognized proliferation-associated gene cyclin D1. RT-PCR and Western blotting were performed to determine the cyclin D1 mRNA and protein levels after treated with the rat medicated sera containing WYHZTL in fibroblasts. As shown in Fig. 4, treated with the rat medicated sera containing WYHZTL for 24, 48 and 72 h led to a down-regulation of cyclin D1 mRNA and protein expression compared with that in the control group (*P* < 0.01), in accordance with the effect of XAV939 known to have inhibition action on cyclin D1 (*P* > 0.05). These results indicated that the sera inhibited proliferation in fibroblasts through down-regulation of cyclin D1 in mRNA and protein level.

### Effect of rat medicated sera containing WYHZTL on survivin in fibroblasts

**Fig. 5:**
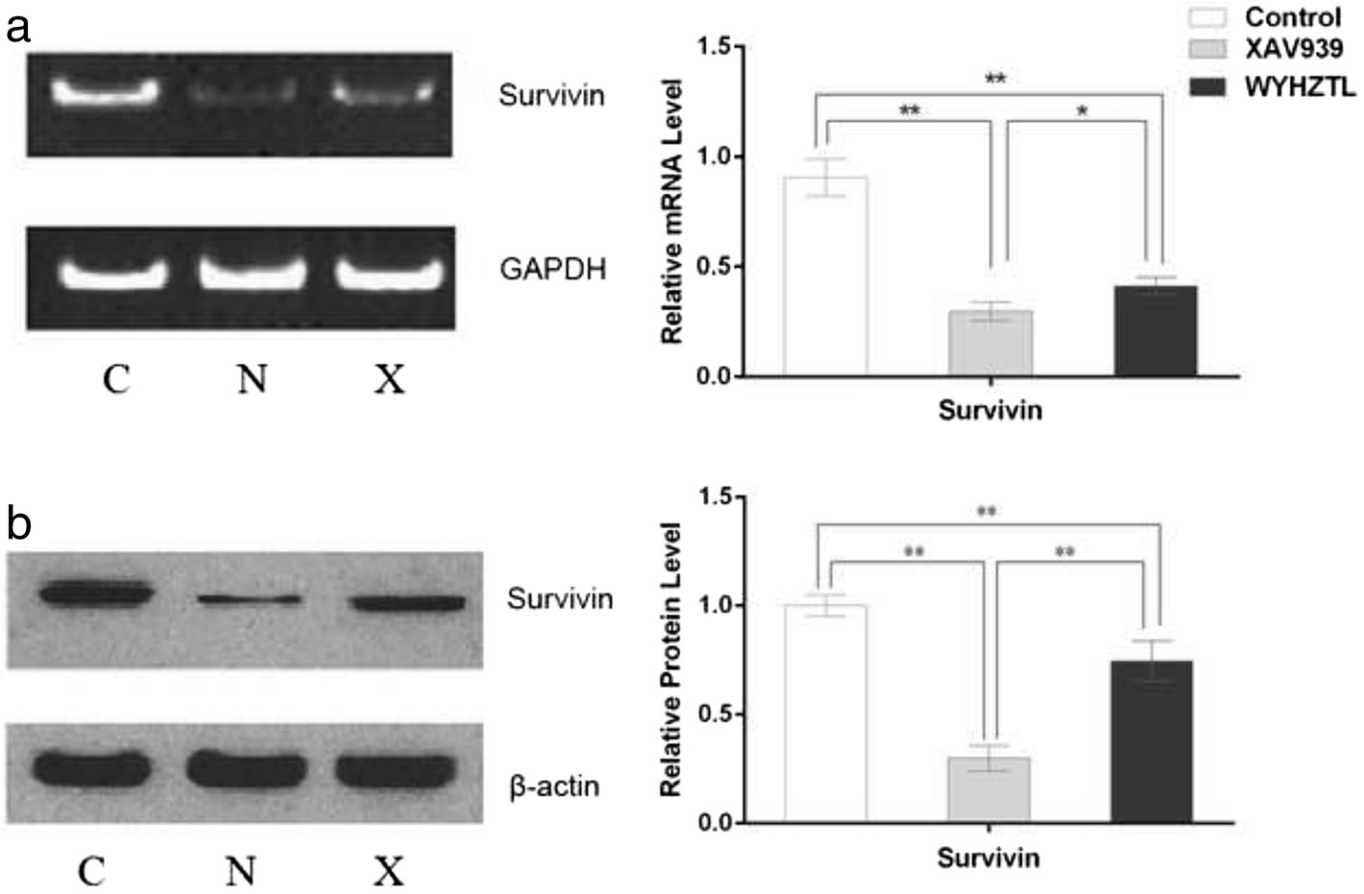
Effect of down-regulation of survivin by the rat medicated sera containing WYHZTL in fibroblasts. **a** The mRNA levels of survivin were detected by RT-PCR after treated with the sera containing WYHZTL for 48 h. GAPDH was applied as an endogenous control and densitometric values were normalized by GAPDH. **b** The protein levels of survivin were detected by western blotting after treated with the sera containing WYHZTL for 48 h. β-actin was used as an endogenous control and densitometric values were normalized by β-actin. C represents control group, N represents XAV939 inhibitor group, X represents rat medicated sera containing WYHZTL group. Representative results are shown. Densitometric values are shown as mean ± SD. Left panels represent RT-PCR/Western blotting gel results, right panels represent bar graphs for relative mRNA and protein levels for survivin. *P < 0.05, **P < 0.01. The results are the mean ± SD of at least 5 independent experiments

The role of the rat medicated sera containing WYHZTL on the recognized apoptosis-associated gene survivin was examined by RT-PCR and Western blotting in fibroblasts. As shown in Fig. 5, treating with the rat medicated sera containing WYHZTL led to a down-regulation of survivin mRNA and protein expression compared to the control group (*P* < 0.01). The sera had a similar inhibitory effect on fibroblasts to XAV939 although the former is weaker than the later (*P* > 0.05). These results indicated that the sera promoted apoptosis in fibroblasts via down-regulation of survivin in both mRNA and protein levels.

## Discussion

SSc is a chronic fibrosing connective tissue disease characterized by a higher mortality rate compared with other connective tissue diseases. In early stages of SSc, the main pathological manifestations are perivascular inflammatory infiltrates and a reduced capillary density, whereas later stages are fibrosis characterized by an excessive accumulation of collagen-rich ECM. The fibrosis disrupts the physiological structure of the affected tissues and interferes with proper organ function. Tissue fibrosis is the primary cause of SSc-related mortality presently and the 10-year survival rate is as low as 54 % - 66 % [21]. There is a high unmet clinical need for effective antifibrotic therapies in SSc, but no drugs have been clearly shown to be effective in reducing specifically the development of SSc fibrosis in clinic up to now [4, 22].

Chinese medical treatment is aimed at adjusting the environmental and human influences through the use of TCM, which exerts a comprehensive effect on the diseases; because the decoction is usually composed of several herbs or minerals according to the symptoms of patients. WYHZTL formula is composed of 10 herbs, among which *Radix Astragali membranacei* exerts an anti-fibrotic effect in rats induced by porcine serum via down-regulating PDGFR-β, inhibiting hepatic stellate cells proliferation and MAPK activation [23]. One of the isolated constituents from *Herba Glechomae longitubae*, asiatic acid, inhibits TGF-β1-induced collagen and PAI-1 expression in keloid fibroblasts through PPAR-γ activation [24]; *Radix Dioscoreae oppositae* attenuats CCl (4)-induced hepatic fibrosis in rats in a dose-dependent manner and the attenuation may be related to the antioxidant properties of *Radix Dioscoreae oppositae* [25]. The ethanolic extract of *Capparis zeylanica Linn* was reported having anti-fibrotic effect via inhibiting the fibroblast proliferation and type I collagen production in SSc [26]. However, the therapeutic action of a TCM formula is far more than that of the total sum of its components, it’s a very complicated process in vivo. TCM formulas prescribed by doctors of Chinese medicine are selected on the basis of the past experience with treating diseases and on the basis of the current health status of the individual patient. Not only do the symptoms of SSc vary from individual to individual, but also the health histories (such as coexisting diseases or syndromes) are different. These factors must be accounted in determining suitable prescriptions. Thus, TCM approach is a comprehensive treatment based on individual needs.

Known abnormalities in SSc that relate to the fibrotic response include fibroblast proliferation increased and apoptosis decreased [27]. So one of treatment strategies for fibrosis in SSc is to inhibit proliferation and promote apoptosis. Cyclins play an important role in cell progression of fibrosis, so the mRNA and protein levels of cell cycle regulatory factor, cyclin D1, were examined. Our results showed that incubation with WYHZTL formula in fibroblasts caused a marked reduction of the mRNA and protein levels of cyclin D1, indicating its crucial for the G1 to S transition [12, 13] and suppression of apoptosis [28]. Marsillach et al. reported that survivin expression is significantly increased during the development of fibrosis [29]. Consistently, Sisson et al. reported that inhibition of survivin restores susceptibility of fibroblasts to Fas-mediated apoptosis. Therefore, survivin may represent a potential target for anti-fibrotic therapies [30]. Additionally, survivin is critically required for suppression of apoptosis and ensuring normal cell division in the G2/M phase of the cell cycle [15]. Our results showed that WYHZTL formula incubation in fibroblasts also caused significant reduction of the mRNA and protein levels of survivin. We have reported that WYHZTL formula has the effect of inhibition on proliferation of SSc skin fibroblasts via blocking the cell cycle transition from the G1 to S phase previously [11]. Therefore, our study suggested that WYHZTL formula arrested the cell cycle in the phase by inhibiting cyclin D1 expression, and its anti-fibrosis effect related to inhibition of cylcin D1 and survivin.

XAV-939 is screened out as an inhibitor of Wnt/β-catenin pathway and demonstrates to stabilize the axin levels through inhibiting tankyrases, and consequently inhibits the Wnt signaling and the expression of target genes, including survivin and cyclin D1 [31]. Aberrant Wnt/β-catenin signaling pathway due to gain of β-catenin function, induces constitutive transcription of cyclin D1 [28, 32]. Our results showed that the effect of WYHZTL formula on survivin and cyclin D1 is consistent with that of XAV939, this further proves that the anti-fibrosis effect of the formula is related to the inhibition of cylcin D1 and survivin.

## Conclusions

In summary, our study demonstrates the WYHZTL formula has antiproliferative and pro-apoptotic actions on fibroblasts in SSc. It also provides valuable information regarding the mechanism of repression of SSc fibrosis, and the effect may be related to the down-regulation of mRNA and protein levels of cyclin D1 and survivin in SSc. Nevertheless, further studies on the other possible mechanisms of anti-fibrosis effect of WYHZTL formula and on the actual active ingredients and/or biotransformed ingredients contributed to the anti-fibrosis effect of WYHZTL are needed.

## Abbreviations

ECM: excessive accumulation of extracellular matrix
I CTP: cross-linked carboxyterminal telopeptide of type I collagen
MAPK: mitogen-activated protein kinase
PAI-1: plasminogen activator inhibitor-1
PDGFR-β: platelet-derived growth factor receptor-β
PIIINP: aminoterminal propeptide of type III procollagen
PPAR-γ: peroxisome proliferator-activated receptor-γ
SSc: systemic sclerosis
TCM: traditional Chinese medicine
TGF-β: transforming growth factor beta
vWF: von Willebrand factor
WYHZTL: Wenyang Huazhuo Tongluo formula
